# Fe_3_O_4_@β-cyclodextrin Nanosystem: A Promising Adjuvant Approach in Cancer Treatment

**DOI:** 10.3390/nano15151192

**Published:** 2025-08-04

**Authors:** Claudia Geanina Watz, Ciprian-Valentin Mihali, Camelia Oprean, Lavinia Krauss Maldea, Calin Adrian Tatu, Mirela Nicolov, Ioan-Ovidiu Sîrbu, Cristina A. Dehelean, Vlad Socoliuc, Elena-Alina Moacă

**Affiliations:** 1 Department of Pharmaceutical Physics, Faculty of Pharmacy, “Victor Babes” University of Medicine and Pharmacy, 300041 Timisoara, Romania; farcas.claudia@umft.ro (C.G.W.); maldea.lavinia@gmail.com (L.K.M.); mirelanicolov@umft.ro (M.N.); 2 Center for Drug Data Analysis, Cheminformatics and the Internet of Medical Things, ”Victor Babes” University of Medicine and Pharmacy, 300041 Timisoara, Romania; 3 Research Center for Pharmaco-Toxicological Evaluation, “Victor Babes” University of Medicine and Pharmacy, 300041 Timisoara, Romania; cadehelean@umft.ro (C.A.D.); alina.moaca@umft.ro (E.-A.M.); 4 Department of Life Sciences, Faculty of Medicine, Vasile Goldis Western University of Arad, 86 No., Liviu Rebreanu St., 310414 Arad, Romania; mihali.ciprian@uvvg.ro; 5 Molecular Research Department, Research and Development Station for Bovine, 32 No., Bodrogului St., 310059 Arad, Romania; 6 Department of Drug Analysis, Chemistry of Environmental Factors, Hygiene, Nutrition, “Victor Babeş” University of Medicine and Pharmacy Timisoara, 2 Eftimie Murgu Sq., 300041 Timisoara, Romania; 7 OncoGen Center for Gene and Cellular Therapies in the Treatment of Cancer, “Pius Brînzeu” County Clinical Emergency Hospital, Timișoara, Blvd. Liviu Rebreanu 156, 300723 Timisoara, Romania; tatu.calin@umft.ro; 8 Department of Immunology and Allergology, Biology, “Victor Babes” University of Medicine and Pharmacy, 300041 Timisoara, Romania; 9 Department of Biochemistry, Faculty of Medicine, “Victor Babes” University of Medicine and Pharmacy Timisoara, 300041 Timisoara, Romania; ovidiu.sirbu@umft.ro; 10 Complex Network Science Center, “Victor Babes” University of Medicine and Pharmacy Timisoara, 300041 Timisoara, Romania; 11 Department of Toxicology, Drug Industry, Management and Legislation, Faculty of Pharmacy, “Victor Babes” University of Medicine and Pharmacy, 300041 Timisoara, Romania; 12 Laboratory of Magnetic Fluids, Center for Fundamental and Advanced Technical Research (CFATR), Romanian Academy—Timisoara Branch, 24 No., Mihai Viteazul Ave., 300223 Timisoara, Romania; vsocoliuc@acad-tim.tm.edu.ro; 13 Research Center for Complex Fluid Systems Engineering, Politehnica University of Timisoara, 1 No., Mihai Viteazu Ave., 300222 Timisoara, Romania

**Keywords:** Fe_3_O_4_@β-CD, HaCaT, A375, adjuvant cancer therapy

## Abstract

The high incidence of melanoma leading to a poor prognosis rate endorses the development of alternative and innovative approaches in the treatment of melanoma. Therefore, the present study aims to develop and characterize, in terms of physicochemical features and biological impact, an aqueous suspension of magnetite (Fe_3_O_4_) coated with β-cyclodextrin (Fe_3_O_4_@β-CD) as a potential innovative alternative nanosystem for melanoma therapy. The nanosystem exhibited physicochemical characteristics suitable for biological applications, revealing a successful complexation of Fe_3_O_4_ NPs with β-CD and an average size of 18.1 ± 2.1 nm. In addition, the in vitro evaluations revealed that the newly developed nanosystem presented high biocompatibility on a human keratinocyte (HaCaT) monolayer and selective antiproliferative activity on amelanotic human melanoma (A375) cells, inducing early apoptosis features when concentrations of 10, 15, and 20 μg/mL were employed for 48 h and 72 h. Collectively, the Fe_3_O_4_@β-CD nanosystem reveals promising features for an adjuvant approach in melanoma treatment, mainly due to its β-cyclodextrin coating, thus endorsing a potential co-loading of therapeutic drugs. Furthermore, the intrinsic magnetic core of Fe_3_O_4_ NPs supports the magnetically based cancer treatment strategies.

## 1. Introduction

One of the types of neoplasms that is increasingly affecting developed countries is melanoma, which, in the United States, ranks fifth in terms of cancer diagnosis frequency [1]. The prognosis is also unfavorable, with melanoma accounting for over 80% of all deaths caused by skin cancers [1,2]. Therefore, for this aggressive tumor, which originates from the melanocytes in the basal layer of the epidermis and easily metastasizes, there is a constant need for the development of new technologies that can improve early diagnosis rates and survival outcomes [3]. Despite the antiproliferative potential of currently used drugs against melanoma, their efficacy remains limited due to systemic toxicity and poor water solubility [4]. Nanotechnology emerges as an innovative and promising tool, actively explored in recent research for the detection and treatment of melanoma [5]. Thanks to their small size and specific surface properties, nanomaterials facilitate the crossing of biological barriers by drugs that are encapsulated or loaded in/on nanoscale structures, where they can effectively exert cytotoxic effects. Moreover, nanosystems offer protection against rapid biodegradation of the active substance, premature elimination from the body, and may also extend plasma half-life, allowing for lower doses to be administered [2,6].

Within the extensive range of nanoparticles utilized in medicine, magnetic iron oxide nanoparticles are particularly notable for their distinctive characteristics, such as biocompatibility, superparamagnetism, biodegradability, extensive surface area, aqueous stability, and straightforward synthesis methods [7].

Magnetite (Fe_3_O_4_), a black ferromagnetic mineral composed of both Fe(II) and Fe(III), is among the most extensively studied iron oxides in nanotechnology applications, particularly due to various biocompatible agents used to customize its surface properties, such as dextran, oleic acid, etc. [4,7,8], and especially beta-cyclodextrin (β-CD) in the context of the present article. The coating (through binding, adsorption, or covalent attachment) with small molecules, organic or inorganic species, either before or after the synthesis process, has the purpose of preventing magnetic nanoparticles from agglomerating as a result of interaction with a biological system, acting as a shield that improves targeting efficiency [9,10].

Cyclodextrins, which are natural cyclic oligosaccharide molecules (derived from starch), have seen a growing interest in research over the past two decades due to their ability to transport active ingredients for the development of topical formulations in the pharmaceutical and cosmetic industries [11,12]. They also improve organoleptic characteristics (masking unpleasant odors, stabilizing fragrance compounds, and protecting against light-induced degradation [13]), as well as enhancing tolerance, solubility, stability, and the controlled release of active substances into the skin [14]. Beta-cyclodextrin, a major cyclodextrin derivative, is a polymer with a hydrophilic outer surface and a hydrophobic inner cavity. It is biodegradable and non-toxic and has been studied for its drug encapsulation properties, including for compounds with anticancer activity [12,15,16].

Interest in evaluating magnetic nanoparticles has continued over the years, especially due to their wide range of applications, such as biomedical employment or commercial products, showing promising benefits. However, the use of magnetic nanoparticles involves significant challenges caused by toxicity, degradation, and elimination from the body, with high costs to ensure stability and industrial-scale production; even data on their possible interactions with healthy tissues and their in vivo and in vitro effects remain relatively limited [17,18]. Thus, the safety profile of magnetic nanoparticles remains a highly debated topic, as their interaction with the microenvironment is influenced by the increased positive surface charge, small size, toxicity dependent on various routes of administration and dosage, as well as the ability of nanoformulations to enhance the concentration of active ingredients absorbed into the bloodstream [19,20].

The present article aims to provide new information regarding Fe_3_O_4_ nanoparticles coated with a β-cyclodextrin shell (further referred to as Fe_3_O_4_@β-CD), thus allowing them to be well dispersed in aqueous medium and to form a stable suspension. The newly developed suspension was assessed in terms of biocompatibility and cytotoxicity on human keratinocytes (HaCaT) and human melanoma cell cultures (A375), respectively. The in vitro results revealed no cytotoxicity on HaCaT cells; thus, the developed suspension may be considered a promising tool for alternative cancer therapy, such as hyperthermic treatment of tumorigenic cells.

## 2. Materials and Methods

### 2.1. Experimental Protocol of the Complexation of Fe_3_O_4_ Nanoparticles with Beta-Cyclodextrin (β-CD)

To develop a comprehensive experimental protocol for the complexation of Fe_3_O_4_ nanoparticles with beta-cyclodextrin (β-CD), it is essential to outline various phases of the procedure, including the synthesis of Fe_3_O_4_ nanoparticles, the preparation of β-CD, and the complexation process itself. Firstly, the Fe_3_O_4_ nanoparticles were obtained through the combustion method, using iron nitrate nonahydrate (Fe(NO_3_)_3_·9H_2_O) as the oxidizing agent and glucose (D-(+)-C_6_H_12_O_6_) as the fuel, in the absence of air. The detailed protocol for synthesizing Fe_3_O_4_ nanoparticles, as well as their characterization, was reported in previous research by our team [21]. Furthermore, the Fe_3_O_4_ nanoparticles obtained were employed to synthesize the nanosystem Fe_3_O_4_@β-CD. Briefly, 50 mg of Fe_3_O_4_ NPs was mixed with an aqueous solution of 18 mg/mL β-CD, in a ratio of 1:2 (Fe_3_O_4_:β-CD), under controlled conditions (constant temperature 22 ± 0.5 °C and pH = 7 ± 0.5) and left to magnetic stirring for 24 h. After that, to enhance interaction, meaning to allow the β-CD to encapsulate portions of the Fe_3_O_4_ nanoparticles, the mixture was subjected to the sonication process (at an amplitude of 50%, using a UP200S from Hielscher Ultrasonics GmbH, Teltow, Germany) for 4 h. After the complexation process, the Fe_3_O_4_@β-CD nanosystem obtained was washed three times with distilled water and separated from the solution using a neodymium block magnet (Q-60-30-15-N, Webcraft GmbH, Gottmadingen, Germany). After that, the Fe_3_O_4_@β-CD nanoparticles were dried at 45 °C in a POL-EKO oven (Wodzisław Slaski, Poland), under an air atmosphere.

### 2.2. Physicochemical Characterization

The nanosystem Fe_3_O_4_@β-CD obtained was subjected to physicochemical characterization to confirm the formation of the complex. The Fourier Transform Infrared Spectroscopy (FTIR) technique allowed for the identification of the characteristic absorption bands associated with the molecular structure of β-CD. Moreover, scanning electron microscopy (SEM) and transmission electron microscopy (TEM) techniques provide insights into the morphological changes that occur post-complexation, as well as insights into particle size distribution. The aqueous colloidal particle size of the nanosystem was measured through photon correlation spectroscopy at room temperature (23 ± 0.5 °C). Through the elemental analysis (energy-dispersive X-ray spectroscopy (EDX)), the presence of chemical elements, as well as their atomic/weight relative percent in the complex, was investigated.

The FTIR spectra of β-CD as well as the Fe_3_O_4_@β-CD nanosystem were obtained using a Shimadzu Prestige-21 spectrometer, from Duisburg, Germany, using KBr pellets. The samples were analyzed in the range 400–4000 cm^−1^, at a resolution of 4 cm^−1^.

SEM analysis was carried out using an FEI Quanta 250 microscope (Eindhoven, The Netherlands), equipped with an ETD (Everhart–Thornley detector for secondary electrons). The work parameters were HV mode, 30 kV, at various magnification orders, for a general overview (100×) and higher surface topography (16,000×) for interest regions. Carbon was used for better conductivity, in a thin layer of 4 nm/deposition, using an AutoAgar Sputter Coater (Agar Scientific Ltd., Essex, UK). The EDX analysis was carried out with an EDAX system (Apollo X detector, Ametek, Mahwah, NJ, USA). The FEI Tecnai 12 Biotwin (Hillsboro, OR, USA) electron microscope was used for TEM analysis, and the Zetasizer Nano ZS (Malvern Instruments, Worcestershire, UK) was used for the determination of the hydrodynamic diameter, H_d_, and polydispersity index, PDI, of the Fe_3_O_4_@β-CD colloidal particles.

### 2.3. Cell Culture

The HaCaT (immortalized human keratinocytes; catalog no. 300493, CLS Cell Lines Service GmbH) cell line and A375 (amelanotic human malignant melanoma; code no. CRL-1619, American Type Culture Collection-ATCC) cells were cultured in DMEM (Dulbecco’s Modified Eagle Medium; Gibco; Paisley, UK) containing 4.5 g/L D-Glucose and L-glutamine. The medium was supplemented with 10% FBS (fetal bovine serum, Gibco; Paisley, UK) with 1% penicillin–streptomycin (Pen/Strep, 10,000 IU/mL; PromoCell, Heidelberg, Germany). The cells were kept under standard conditions (humidified atmosphere, 5% CO_2_, and 37 °C) and passaged every third day. Cell number was determined by using the cell counting chamber (Neubauer) in the presence of Trypan blue.

### 2.4. Stock Solution

A stock solution of 8.84 mg/mL was obtained and was stored at −20 °C. The final concentrations were prepared by successive dilution of the stock solution with the growth medium, obtaining solutions of 10, 15, and 20 µg/mL, respectively.

### 2.5. Alamar Blue Colorimetric Test

The viability of HaCaT and A375 cells was assessed through the Alamar Blue assay. In brief, the cells were seeded onto 96-well plates (1 × 10^4^ cells/200 µL media/well) and treated with selected concentrations (10, 15, and 20 µg/mL) of the Fe_3_O_4_@β-CD suspension for 48 and 72 h, respectively. Afterwards, Alamar Blue reagent was added in each well, and after a minimum of 3 h, the absorbance was determined using wavelengths of 570 and 600 nm (xMark™ Microplate Spectrophotometer—BioRad Laboratories, Inc., Thermo Fischer Scientific, Carlsbad, CA, USA). The cellular viability was quantified using a well-known formula described in a previous study [22].

### 2.6. DAPI (4′,6-Diamidino-2-Phenylindole) Staining

Detection of the cells’ nuclear morphological aspect was assessed through nuclear DAPI staining. In brief, the protocol consisted of seeding both cell lines (HaCaT and A375) on coverslips using 6-well plates. Afterwards, the cells were treated for 72 h with three selected concentrations (10, 15, and 20 µg/mL) of the Fe_3_O_4_@β-CD suspension. DAPI staining was performed as previously described [23,24], following the next steps: fixation with 4% paraformaldehyde in 1xPBS, permeabilization with 2% Triton X-100, blocking by using 30% FCS in 0.01% Triton X-100, and staining with 4′,6′-diamidino-2-phenylindole (Sigma-Aldrich) for 15 min in a dark chamber. Cell nuclei were visualized at a magnification of 20× using the Olympus IX73 inverted fluorescence microscope (Olympus, Tokyo, Japan).

### 2.7. Annexin V/PI Assay for the Quantification of Apoptosis and Necrosis

To assess the early and late stages of apoptosis, as well as necrosis induced by selected concentrations (10, 15, and 20 µg/mL) of the investigated formulation, an assay based on Annexin V and propidium iodide (PI) labeling was performed. A total of 5 × 10^5^ cells/well were seeded into 6-well plates (Greiner Bio-One, GmbH, Germany) and incubated overnight to allow for cell adherence to the bottom surface. Following a 24 h incubation period, the culture medium was replaced with fresh medium containing the tested formulation. Untreated cells served as a negative control, and cells treated with DMSO served as a solvent control. After 72 h of incubation, the cells were trypsinized, and an Annexin V-FITC/propidium iodide (PI) kit (Invitrogen, ThermoFisher, Vienna, Austria) was utilized for apoptosis analysis according to the manufacturer’s instructions. Briefly, cells were washed twice with 1× Annexin V Binding Buffer, centrifuged at 1500 rpm for 5 min, resuspended in the same binding buffer, and incubated with 5 μL of Annexin V-FITC for 10 min at room temperature in the dark. In the subsequent step, cells were washed with 200 μL of binding buffer and centrifuged, and the resulting cell pellet was resuspended in 190 μL of binding buffer, followed by the immediate addition of 10 μL of propidium iodide solution immediately before flow cytometry analysis. Data acquisition was conducted using a FACSCalibur flow cytometer (Becton Dickinson, Franklin Lakes, NJ, USA), and the subsequent data analysis was performed using FCSAlyzer 0.9.22-alpha.

## 3. Results

### 3.1. Physicochemical Investigation of Fe_3_O_4_@β-CD

**Figure 1:**
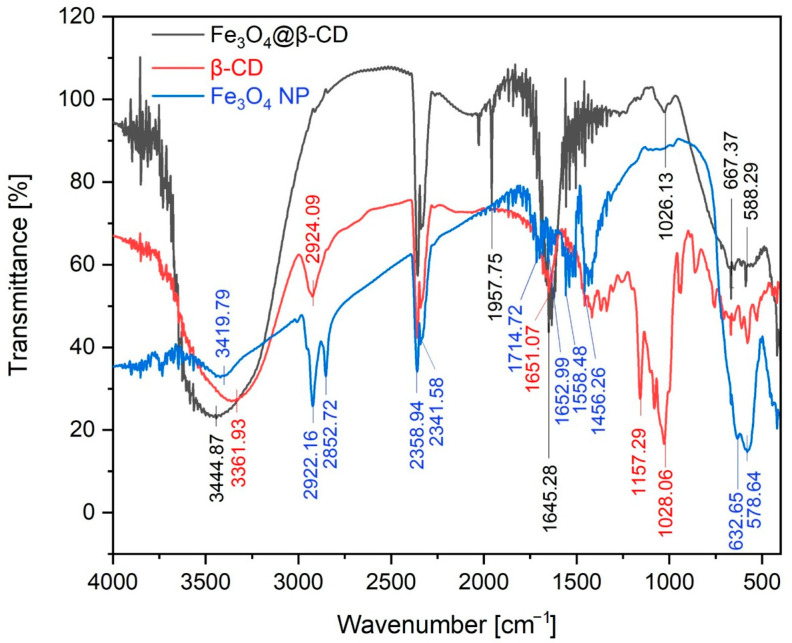
The FTIR spectra for Fe_3_O_4_ NPs (blue line), β-CD (red line), and the Fe_3_O_4_@β-CD nanosystem (black line).

Figure 1 shows the FTIR spectra of Fe_3_O_4_ NPs (blue line), β-CD (red line), as well as of the Fe_3_O_4_@β-CD nanosystem (black line). The most important absorption bands recorded in the case of Fe_3_O_4_ NPs are highlighted on the graphic (blue color). The absorption band at 3419.79 cm^−1^ highlights the O-H stretching vibration group. The asymmetric and symmetric stretching vibrations of the CH_2_ bands are recorded at 2922.16 cm^−1^ and 2852.72 cm^−1^. The multiple bands recorded in the range 1714.72 and 1456.26 cm^−1^ overlay the stretching vibration of the C=C and C-O groups, as well as the bending vibration of the H-O-H and C-H groups.

The β-CD spectrum (red line) usually shows three characteristic bands around the values of 1028, 1157, and 1650 cm^−1^. The bands recorded at the 1028.06 cm^−1^ and 1157.29 cm^−1^ wavenumbers correspond to the asymmetric C-O-C vibrations and coupled C-H/C-O stretching vibrations, as well as to C-H overtone stretching. At 1651.07 cm^−1^, the absorption band specific for the H-O-H deformation of water functional groups present in β-CD is recorded, as well as C=C and -C-O stretching vibration groups. The intense peak recorded at 2924.09 cm^−1^ is assigned to the C-H symmetric/asymmetric stretching band from the β-CD. At 3361.93 cm^−1^ (red line), the O-H stretching absorption band from the hydroxyl group is recorded. After the interaction with Fe_3_O_4_ NPs, this absorption band is shifted to 3444.87 cm^−1^ (black line), increasing its broadening as well.

On the Fe_4_O_4_@β-CD spectrum (black line), at 588.29 cm^−1^, the recorded characteristic absorption bands can be attributed to the presence of the Fe-O bond in which iron is present in tetrahedral coordination. This absorption band was slightly shifted because in the Fe_3_O_4_ NP spectrum, this band was recorded at 578.64 cm^−1^. It can also be noticed that the band located at 1026.13 cm^−1^ is assigned to the C-O stretching vibration.

**Figure 2:**
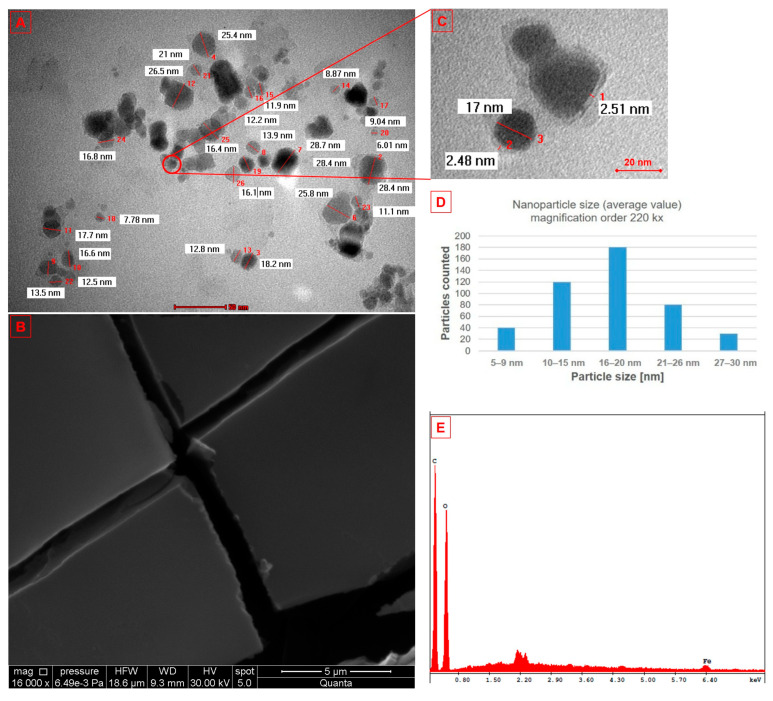
(**A**,**C**) TEM and (**B**) SEM images of Fe_3_O_4_@β-CD, (**D**) particle size distribution, and (**E**) EDX spectrum of Fe_3_O_4_@β-CD nanosystem.

Figure 2 shows the morphology and elemental composition investigations of the Fe_3_O_4_@β-CD nanosystem. From the SEM image (Figure 2B), it can be observed that Fe_3_O_4_ NPs had affinity for the OH groups inside the β-CD but not for those on its surface due to the irregularly shaped, smooth plate-like morphology. If the Fe_3_O_4_ NPs had bound to the OH groups on the surface of the β-CD, then the nanoparticles should have been porous, with an undefined spherical shape. Another confirmation of the fact that Fe_3_O_4_ NPs are inside the hydrophobic core, although the exterior surface of cyclodextrins is somewhat hydrophilic, is represented by the TEM image (Figure 2C), where the β-CD layer could be observed and measured, having about 3 nm.

Regarding the morphology of the nanosystem (Figure 2A), one can observe that the nanoparticles have oblong or rounded shapes, arranged quasi-uniformly, agglomerated, with a narrow size distribution range between 5 and 30 nm, with a mean size of 18.1 ± 2.1 nm, according to ImageJ software (version number 1.53f, https://imagej.nih.gov/ij/, accessed on 2 June 2025), after measuring at least 150 particles. In addition, the elemental composition of the Fe_3_O_4_@β-CD nanosystem, evaluated by EDX analysis (Figure 2E), showed that the C, O, and Fe are the only elements present in the sample, indicating the purity of the nanosystem synthesized. The atomic and weight percent ratios of the identified elements are presented in Table 1.

The dynamic light scattering technique (DLS) showed that the aqueous colloidal nanosystem has a size of 106.26 nm with a zeta potential of—22.31 mV and a polydispersity index (PDI) of 0.186%.

### 3.2. Cell Viability Assessment Through the Alamar Blue Colorimetric Test

As a first-line biosafety assessment, the Alamar Blue assay was employed to evaluate the effects of the Fe_3_O_4_@β-CD suspension on cellular metabolic activity of both normal (HaCaT) and cancerous (A375) cell lines to determine the cytocompatibility of the newly developed suspension.

**Figure 3:**
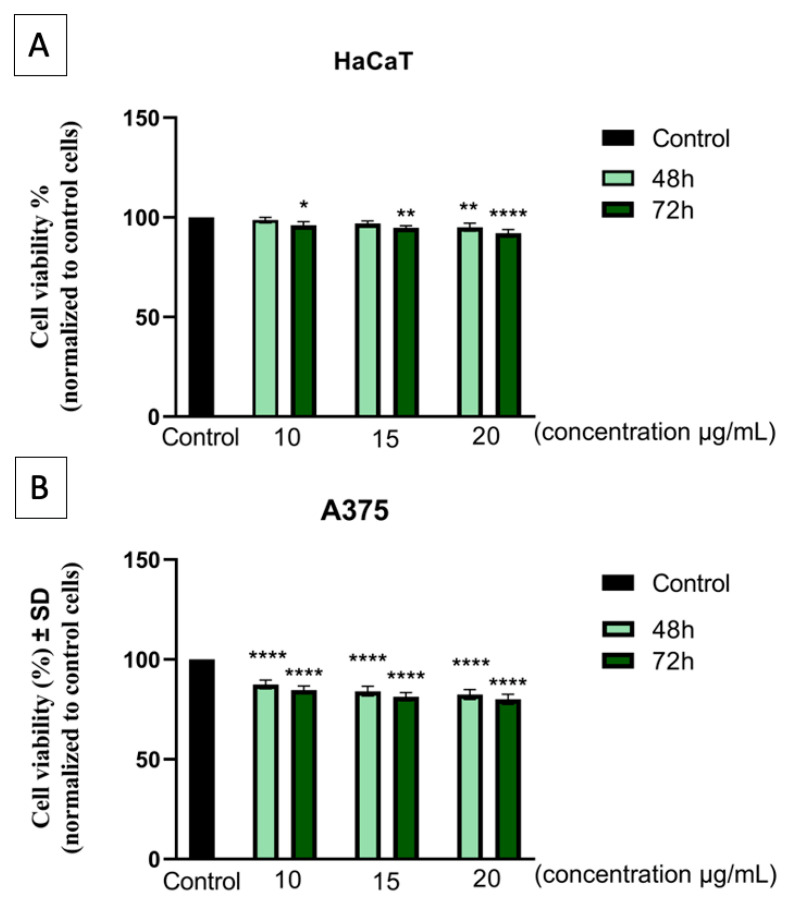
Cell viability assessment on (**A**) human immortalized keratinocytes (HaCaT) and (**B**) human melanoma (A375) after 48 h and 72 h exposure to three different concentrations (10, 15, and 20 μg/mL) of the Fe_3_O_4_@β-CD suspension. Results are presented as the cell viability percentage (%) normalized to control cells (cells treated with medium). The data represent the mean values ± standard deviation (SD) of three independent experiments (*n* = 3). One-way ANOVA analysis and Dunnett’s multiple comparisons test were used to determine the statistical differences between sample-treated and control cells (* *p* < 0.1; ** *p* < 0.01; **** *p* < 0.0001).

As presented in Figure 3A, the non-tumorigenic cell line of human keratinocytes (HaCaT) did not cause a significant decrease in cell viability when exposed to concentrations of 10, 15, and 20 μg/mL of the Fe_3_O_4_@β-CD suspension for an interval of 48 h and 72 h. HaCaT cells expressed viabilities between 98.63% and 95.11% at 48 h post-exposure to the Fe_3_O_4_@β-CD suspension, depending on the dose concentration, while at 72 h post-treatment, cell viability ranged from 96.12% to 92.02% in a dose-dependent manner.

Human melanoma cells (A375) presented a slightly more affected cell population (Figure 3B), with cell viabilities of 87.45%, 84.12%, and 82.44% after a 48 h treatment with concentrations of 10, 15, and 20 μg/mL of the Fe_3_O_4_@β-CD suspension, respectively. Cell viability rates decreased at 72 h post-exposure, obtaining viabilities of 84.72%, 81.36%, and 80.07% following a dose-dependent pattern.

### 3.3. DAPI Staining

Since the Alamar blue assay revealed no significant cytotoxicity on HaCaT cells and only a slight reduction in viability on A375 melanoma cells following treatment with Fe_3_O_4_@β-CD suspension, it became necessary to explore whether these effects were associated with early nuclear changes not captured by metabolic viability alone. Therefore, DAPI staining was performed to examine potential nuclear condensation or fragmentation, indicative of apoptotic processes [25,26]. This morphological assessment complements the metabolic data and helps clarify whether subtle cytotoxic effects may be linked to apoptosis initiation.

**Figure 4:**
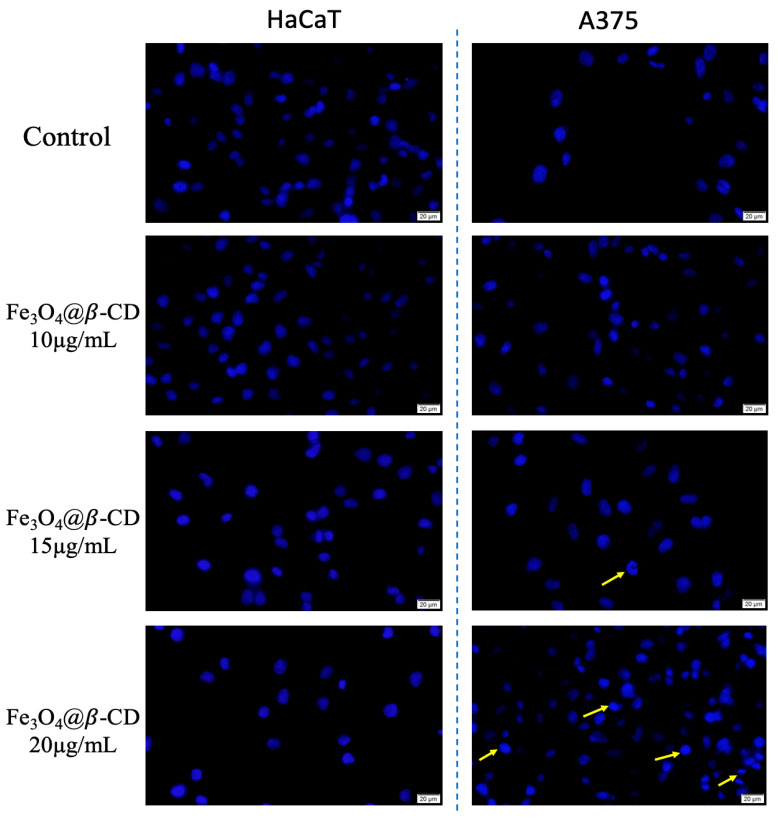
Morphological aspects of human keratinocytes (HaCaT) and human melanoma (A375) cell line after exposure to three different concentrations (10, 15, and 20 μg/mL) of Fe_3_O_4_@β-CD suspension for an interval of 72 h. Cells treated with specific culture medium were considered control cells. Signs of apoptosis are marked with yellow arrows.

As presented in Figure 4, DAPI staining revealed no significant nuclear alterations in human keratinocytes treated with the Fe_3_O_4_@β-CD suspension, indicating the absence of notable apoptotic events. However, A375 human melanoma cells exhibited early morphological signs of apoptosis. At a concentration of 15 µg/mL, some cells displayed membrane blebbing (yellow arrow), while treatment with 20 µg/mL led to chromatin condensation (yellow arrows).

### 3.4. Annexin V/PI Assay

While DAPI staining provided visual evidence of potential nuclear alterations in HaCaT and A375 cells treated with the Fe_3_O_4_@β-CD, this method alone does not differentiate between apoptotic and necrotic pathways. To further characterize the nature of cell death and to quantify early and late apoptotic events, we performed Annexin V/propidium iodide (PI) staining followed by flow cytometric analysis.

**Figure 5:**
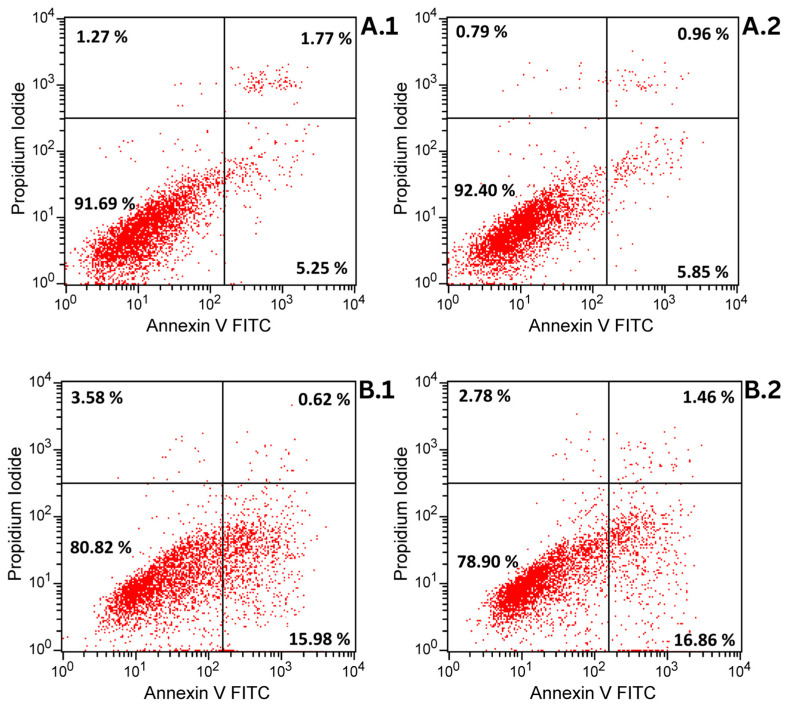
Dot plot of Annexin V/PI assay: (**A.1**) HaCaT cells control (untreated); (**A.2**) HaCaT cells treated with Fe_3_O_4_@β-CD 10 µg/mL; (**B.1**) A375 cells control (untreated); (**B.2**) A375 cells treated with Fe_3_O_4_@β-CD 10 µg/mL.

Table 2 and Figure 5 show the results of Annexin V/PI analysis. The Fe_3_O_4_@β-CD complex showed no toxic effects on any cell line tested. In human keratinocytes (HaCaT), cell viability remained consistently high (91–92%) across all concentrations (10–20 µg/mL), comparable to untreated controls (untreated cells; *p* > 0.05), confirming the compound’s lack of cytotoxicity. However, the results observed in A375 human melanoma cells showed a rate of 78–83% viability.

Regarding the effect on apoptosis, in HaCaT keratinocytes, late apoptosis slightly decreased at 10 µg/mL (from 1.77% to 0.96%, *p* = 0.038). In contrast, A375 melanoma cells exhibited a very small but statistically significant increase in late apoptosis at the same concentration (from 0.62% to 1.46%, *p* = 0.004).

Necrosis remained stable across all cell lines after treatment (HaCaT: 1.2–1.3%; A375: 2.3–3.6%), with no significant differences compared to the controls (untreated cells; *p* > 0.05). Notably, the effects did not exhibit concentration dependence: neither higher doses (15–20 µg/mL) nor prolonged exposure (presented results are after 72 h treatment) altered apoptosis or viability beyond the changes observed at 10 µg/mL.

## 4. Discussion

At the skin level, it has been shown that the homeostasis of healthy cells exposed long-term to pollutants and UV radiation can be disrupted, leading to inflammation and oxidative stress through the release of reactive oxygen species. These, in turn, trigger excessive melanin production, contributing to the appearance of pigmentation disorders, such as freckles, melasma, and solar lentigo, up to melanoma [27,28]. Moreover, the skin also represents the main route of nanoparticle penetration into the body, according to the extensive study conducted by Vance and his collaborators, which analyzed 770 products based on nanotechnology [29]. Among these nanoparticles, magnetite nanoparticles (Fe_3_O_4_ NPs) have become increasingly used in the cosmetic industry in recent years. They are valued for their antioxidant, detoxifying, and skin-protective properties, acting to capture and neutralize free radicals, thereby reducing oxidative stress and pigmentation disorders. Additionally, Fe_3_O_4_ NPs can transport and release active substances in a controlled manner and, under the influence of external stimuli such as magnetic fields, can enhance skin penetration and distribution of these substances [30]. Regarding the action of Fe_3_O_4_ NPs on neoplastic cells, due to their nanoscale diameter (20–100 nm) and strong magnetism, they have been frequently used as hyperthermia agents capable of generating intense heating (45–46 °C) following the application of magnetic alternative fields, thereby destroying activated tumor cells, while minimizing unwanted side effects in the rest of the body [31,32,33,34]. Our research group reported a study conducted under hyperthermic conditions, aimed at assessing the action of iron oxide nanoparticles, emphasizing that the viability of the healthy cell line was not affected (at a concentration of 30 µg/mL), whereas the neoplastic cell line (MDA-MB-231 breast adenocarcinoma) showed a significant decrease in tumorigenic viability [35]. Similar results are supported by another study investigating the effects of Fe_3_O_4_-PA-(HP-γ-CDs) on the HaCaT cell line under both normal conditions (37 °C) and hyperthermia (42.5 °C), with no changes observed in healthy cell viability over 24 h in either case [36].

Regarding β-CD utilization, an enhanced antiproliferative effect has been observed when imiquimod nanoparticles were incorporated into β-CD, leading to increased biocompatibility of drug delivery systems by ensuring improved bioavailability, solubility, and permeability, as well as more controlled and prolonged release, protected from premature degradation [37]. Another study investigated the complexation of the natural alkaloid “harman” with β-CD, which resulted in amplified cytotoxicity against melanoma (A2058 cell line) by accelerating apoptosis [38]. Therefore, to increase Fe_3_O_4_ stability and biocompatibility, we developed herein the Fe_3_O_4_@β-CD suspension.

The complexation of Fe_3_O_4_ nanoparticles with β-CD has garnered attention in various fields such as catalysis, drug delivery, and environmental remediation. The incorporation of β-CD provides structural and functional benefits due to its hydrophilic exterior and hydrophobic cavity, facilitating the interaction with Fe_3_O_4_ NPs. The Fe_3_O_4_ NPs used in the present study showed superparamagnetic properties, high surface area, and small particle size (results published in reference [21]), making them ideal candidates for numerous applications, including drug delivery.

The interaction between β-CD and Fe_3_O_4_ nanoparticles takes place through the adsorption process, facilitated by the strong hydrogen bonding capacity, which occurs between the hydroxyl groups present inside the β-CD cavity and the hydroxyl groups on the surface of the Fe_3_O_4_ NPs, under certain conditions [39,40]. Through such interactions, stable, soluble, and functional complexes can be formed in aqueous medium. Ebrahimiasl et al. confirm that the coating of magnetite nanoparticles is significantly influenced by hydrogen bonding; hence, a stable and reusable system is obtained [39]. Due to the fact that the interaction between nanoparticles and β-CD is a non-covalent interaction, the reversible mechanism can take place, i.e., the release of β-CD from the nanoparticle surface. This can only happen under specific conditions (temperature and pH) and is particularly advantageous in drug delivery systems that allow controlled release into solution [41].

After the complexation process, it is crucial to characterize the resultant nanosystem to confirm the formation of the Fe_3_O_4_@β-CD complex. The physicochemical investigations, in terms of FTIR spectroscopy, indicate successful complexation through the shifting of characteristic peaks in Fe_3_O_4_@β-CD with respect to pure β-CD and naked Fe_3_O_4_ NP, confirming a strong binding with the Fe-O bond [42,43]. Based on the literature data, the FTIR spectrum of naked Fe_3_O_4_ NP exhibits a strong absorption band around 570 cm^−1^ (in our case, this band was recorded at 578.64 cm^−1^) [44,45]. This band is assigned to the Fe-O stretching mode of the tetrahedral and octahedral sites. Our results are in agreement with those reported in the literature [46,47,48,49,50]. Emara et al. [42] reported that FTIR spectroscopy highlights the characteristic peaks for β-CD and Fe_3_O_4_ NPs, confirming complexation and indicating successful incorporation of β-CD onto the nanoparticle surface. According to the authors’ results, also in our case, the peaks corresponding to the magnetite phase, notably between 500 and 600 cm^−1^, were still observable in the nanosystem, indicating that the structural integrity of Fe_3_O_4_ NPs was preserved during the complexation process. Moreover, studies exploring the stabilizing role of β-CD have revealed that the unique donut-like structure of β-CD is effective in preventing agglomeration of nanoparticles, leading to isolated and uniformly distributed particles [51]. Biehler et al. [52] noted that stable, well-dispersed nanoparticles possess enhanced catalytic activities due to their increased surface area, which β-CD properties facilitate by restricting unregulated growth.

The morphological analysis showed that the Fe_3_O_4_@β-CD nanosystem has nanoparticles with oblong or rounded shapes, with a narrow size distribution and a mean of 18.1 nm. The irregularly shaped, smooth plate-like morphology observed in the SEM image reinforces the claim that the absorbing functional groups in the β-CD structure may act as stabilizers or surfactants that chelate with Fe atoms, creating a steric block [47]. Although the size of the aqueous colloidal nanosystem showed a value of 106.22 nm, it remains a potential candidate for biomedical uses, due to its stability and biocompatibility (zeta potential was—22.31 mV and had a PDI of 0.186), as well as the proper shape and size. Electrostatic stability and interactions between particles in a colloid are indicated by the zeta potential. This indicates the magnitude of electrostatic or charge repulsion between particles of the same charge in suspension. Therefore, a stronger electrostatic repulsion leads to a higher absolute zeta potential, i.e., a lower probability that particles in suspension will aggregate. Colloidal suspensions that have absolute zeta potentials greater than ±30 mV are often considered stable due to electrostatic repulsion, which prevents agglomeration of particles [53,54]. In contrast, colloidal suspensions recording zeta potentials between −11 and −20 mV suggest a potential instability and a likelihood of agglomeration of the particles over time, as they are close to the threshold [55,56]. The zeta potential obtained in our case correlates well with the TEM analysis, which indicates that the nanoparticles show a slight agglomeration. Regarding the nanoparticle’s diameter, it has been stated that the size distribution of the NPs plays a major role in their performance, as a narrow size distribution and diameters smaller than 100 nm tends to ensure consistent behavior and fabrication credibility in clinical settings [57], whereas larger particles may be advantageous for imaging and targeted delivery in particular scenarios, such as solid tumors [58]. This interaction between size and biological function is pivotal, as it dictates how nanoparticles can interact with target tissues [59]. Once again, our results are in concordance with those reported in the literature [47,48,51].

One can observe a discrepancy between the TEM diameter (18.1 nm) and the DLS hydrodynamic diameter (106.22 nm). This could be attributed to the fundamental principles underlying these two measurement techniques. Through TEM, a direct measurement of the nanoparticles in the dry state is obtained, i.e., the real diameter of the nanoparticle core. The DLS technique works by analyzing the dynamic scattering of light by nanoparticles suspended in a solution, i.e., measuring the hydrodynamic diameter of a suspension or a colloid [60]. Therefore, by the DLS technique, the whole colloid is measured, consisting of nanoparticles (in our case slightly aggregated), the β-CD layer dimension, and the size of the solvent layer surrounding the nanoparticles (aqueous solution) [61,62]. This hydrated complex contributes to the overall hydrodynamic volume, causing the dimensions obtained by DLS to be considerably larger than those determined by TEM [63,64]. It has been argued that coatings on the surface of nanoparticles, such as lipids or polymers (surfactants) used for stabilization or functionalization, further amplify size differences [64,65]. Moreover, DLS measures an average of the colloidal complex, providing information about the aggregation of nanoparticles that cannot be investigated by TEM evaluation [66,67].

While β-CD-complexed Fe_3_O_4_ nanoparticles show significant promise, ongoing research continuously aims to optimize their properties. Methods, including co-encapsulation strategies with various therapeutic agents or stabilizers, further enhance their utility in biomedical applications as indicated by various studies [51,68,69]. These composite structures not only preserve the unique properties of Fe_3_O_4_ NPs but also provide robust release mechanisms of the drug suited for targeted therapies. As reported in the literature, Fe_3_O_4_@β-CD systems were successfully developed under various formulations for multiple biomedical applications, such as identification agents for cholesterol detection [70,71], as adsorbent tools for the removal of Bisphenol-A in water when Fe_3_O_4_@β-CD systems were embedded onto graphene [72], or for industrial wastewater treatment [73] but also as a drug delivery system when Janus magnetic nanoparticles functionalized with β-CD (Fe_3_O_4_@SiN/β-CD) were employed [74]. However, all the above-mentioned studies used exclusively the coprecipitation method to obtain the Fe_3_O_4_ NPs. The present study is the only one that reports the successful synthesis of a Fe_3_O_4_@β-CD nanosystem using the combustion method to obtain Fe_3_O_4_ NPs, also reporting promising data in terms of biocompatibility and cytotoxicity on human keratinocytes (HaCaT) and human melanoma cell cultures (A375) for the Fe_3_O_4_@β-CD nanosystem.

The biocompatibility of the newly developed Fe_3_O_4_@β-CD suspension was assessed through different in vitro assays. Almar blue colorimetric test revealed that both types of cell cultures (non-tumorigenic cell line HaCaT and tumorigenic A375 cells) showed a concentration-dependent decrease in viability, as well as time–exposure dependence. For the HaCaT cell line, the highest viability of 98.63% was recorded when the culture was treated with 10 μg/mL of the Fe_3_O_4_@β-CD suspension for 48 h, and the lowest viability of 92.02% was observed after 72 h when the concentration of 20 μg/mL was used. Also, in the case of the A375 cell culture, the lowest rate of 80.07% was recorded after 72 h at a concentration of 20 μg/mL.

DAPI staining results provided morphological confirmation of the selective cytotoxic behavior observed in the viability assays. While human keratinocytes (HaCaT cells) retained normal nuclear aspects across all tested concentrations, indicating good biocompatibility of the Fe_3_O_4_@β-CD suspension, A375 melanoma cells exhibited several apoptotic features. Specifically, membrane blebbing was observed at 15 µg/mL, and chromatin condensation became evident at 20 µg/mL, typical aspects of apoptosis. Annexin V/PI analysis reveals the compound’s lack of cytotoxicity on the tested lines, but a minor increase in the percentage of apoptotic or necrotic cells compared to untreated cells was observed in the case of A375 cells. Correlated with the Alamar blue analysis and DAPI staining, it is suggested that the compound exhibits antiproliferative properties and a slight apoptotic index on A375 cells compared to non-tumorigenic cells (HaCaT).

These findings support the hypothesis that the Fe_3_O_4_ nanoparticle suspension may trigger apoptotic pathways in malignant cells with minimal cytotoxicity on healthy tissue, an important consideration for its future application in adjuvant anticancer therapies, such as hyperthermia-based treatments.

## 5. Conclusions

The current study presents the development of an aqueous suspension of magnetite coated with β-cyclodextrin (Fe_3_O_4_@β-CD) as an adjuvant approach to melanoma treatment, reporting for the first time the synthesis of a Fe_3_O_4_@β-CD nanosystem starting from Fe_3_O_4_ NPs obtained through the combustion method. The newly developed nanosystem revealed physicochemical characteristics suitable for biomedical applications, while the biological profile demonstrated good biocompatibility on human keratinocytes (HaCaT cell line), with early apoptosis events on human melanoma cells (A375), without inducing necrosis. Altogether, the nanosystem shows promising features for a further drug loading strategy through its β-cyclodextrin layer, while the magnetic core of the magnetite NPs assures the magnetically based cancer treatments approach of the nanoplatform, thus encouraging the need for further investigations of the nanosystem by developing drug-loaded platforms and employing advanced in vitro models, such as 3D reconstructed human tissues.

## Figures and Tables

**Table 1:** The elemental composition of the Fe_3_O_4_@β-CD nanosystem.

Element	Wt %	At %	K-Ratio
C k	53.15	60.34	0.2886
O k	46.40	39.55	0.0815
Fe k	0.45	0.11	0.0042
Total	100.00	100.00	

**Table 2:** Quantitative analysis of Fe_3_O_4_@β-CD effects on cell populations after 72 h treatment. Data shown as mean ± SD (*n* = 3). Significance vs. control was determined by one-way ANOVA (normal distribution) or Kruskal–Wallis test (non-normal data) with post hoc testing: ** *p* < 0.01, * *p* < 0.05.

Conc. (μg/mL)	Viable Cells (%)	Early Apoptotic Cells (%)	Late Apoptotic Cells (%)	Necrotic Cells (%)
HaCaT				
0	91.69 ± 1.77	5.25 ± 1.39	1.77 ± 0.49	1.27 ± 0.92
10	92.40 ± 4.93	5.85 ± 4.93	0.96 ± 0.27 *	0.79 ± 0.22
15	91.68 ± 5.23	5.92 ± 4.42	1.19 ± 0.44	1.21 ± 0.61
20	91.12 ± 5.28	6.53 ± 4.93	1.18 ± 0.43	1.17 ± 0.43
A375				
0	80.82 ± 6.62	15.98 ± 8.47	0.62 ± 0.16	3.58 ± 2.99
10	78.90 ± 4.52	16.86 ± 2.96	1.46 ± 0.09 **	2.78 ± 2.49
15	83.07 ± 3.89	13.77 ± 4.83	0.90 ± 0.53	2.26 ± 2.06
20	81.85 ± 5.40	14.85 ± 5.11	0.93 ± 0.31	2.39 ± 1.72

## Data Availability

Authors can provide raw data upon request.
